# A murine macrofilaricide pre-clinical screening model for onchocerciasis and lymphatic filariasis

**DOI:** 10.1186/s13071-014-0472-z

**Published:** 2014-10-24

**Authors:** Alice Halliday, Ana F Guimaraes, Hayley E Tyrer, Haelly Mejane Metuge, Chounna Ndongmo Winston Patrick, Kengne-Ouafo Jonas Arnaud, Tayong Dizzle Bita Kwenti, George Forsbrook, Andrew Steven, Darren Cook, Peter Enyong, Samuel Wanji, Mark J Taylor, Joseph D Turner

**Affiliations:** Department of Parasitology, Liverpool School of Tropical Medicine, Liverpool, UK; Research Foundation for Tropical Diseases and the Environment, Buea, Cameroon; Department of Microbiology and Parasitology, Parasite and Vector Research Unit, University of Buea, Buea, Cameroon

**Keywords:** Anti-filarial, Lymphatic filariasis, Onchocerciasis, Macrofilaricide, *Brugia*, *Onchocerca*, *Wolbachia*

## Abstract

**Background:**

New drugs effective against adult filariae (macrofilaricides) would accelerate the elimination of lymphatic filariasis and onchocerciasis. Anti-*Onchocerca* drug development is hampered by the lack of a facile model. We postulated that SCID mice could be developed as a fmacrofilaricide screening model.

**Methods:**

The filaricides: albendazole (ABZ), diethylcarbamazine (DEC), flubendazole (FBZ), ivermectin (IVM) and the anti-*Wolbachia* macrofilaricide, minocycline (MIN) were tested in *Brugia malayi* (*Bm*)-parasitized BALB/c SCID mice *vs* vehicle control (VC). Responses were compared to BALB/c wild type (WT). *Onchocerca ochengi* male worms or onchocercomata were surgically implanted into BALB/c SCID, CB.17 SCID, BALB/c WT mice or *Meriones* gerbils. Survival was evaluated at 7–15 days. BALB/c SCID were tested to evaluate the responsiveness of pre-clinical macrofilaricides FBZ and rifapentine (RIFAP) against male *Onchocerca.*

**Results:**

WT and SCID responded with >95% efficacy following ABZ or DEC treatments against *Bm* larvae (*P < 0.0001*). IVM was partially filaricidal against *Bm* larvae in WT and SCID (WT; 39.8%, *P = 0.0356* and SCID; 56.7%, *P = 0.026*). SCID responded similarly to WT following IVM treatment of microfilaraemias (WT; 79%, *P = 0.0194.* SCID; 76%, *P = 0.0473*). FBZ induced a total macrofilaricidal response against adult *Bm* in WT and SCID (WT; *P = 0.0067,* SCID; *P = 0.0071*). MIN induced a >90% reduction in *Bm Wolbachia* burdens (*P < 0.0001*) and a blockade of microfilarial release (*P = 0.0215*) in SCID. Male *Onchocerca* survival was significantly higher in SCID vs WT mice, but not gerbils, after +15 days (60% *vs* 22% *vs* 39% *P = 0.0475*). Onchocercoma implants had engrafted into host tissues, with evidence of neovascularisation, after +7 days and yielded viable macro/microfilariae *ex vivo*. FBZ induced a macrofilaricidal effect in *Onchocerca* male implanted SCID at +5 weeks (FBZ; 1.67% *vs* VC; 43.81%, *P = 0.0089*). *Wolbachia* loads within male *Onchocerca* were reduced by 99% in implanted SCID receiving RIFAP for +2 weeks.

**Conclusions:**

We have developed a ‘pan-filarial’ small animal research model that is sufficiently robust, with adequate capacity and throughput, to screen existing and future pre-clinical candidate macrofilaricides. Pilot data suggests a murine onchocercoma xenograft model is achievable.

## Background

The tissue dwelling filaria, *Onchocerca volvulus,* infects an estimated 37 million people, predominantly in Sub-Saharan Africa [1]. Ocular onchocerciasis (river blindness) is the second leading cause of worldwide preventable blindness [2] and is a priority neglected tropical disease (NTD) targeted for elimination [3]. Current treatments with ivermectin (IVM) target the transmissive stages of *O. volvulus*, microfilariae (mf), in the skin, with limited efficacy against adult (macrofilariae) fertility or survival [4]. Whilst effective at reducing disease, due to repopulation of the skin with mf post-treatment, IVM has to be administered periodically. In addition, IVM has to be delivered for protracted periods (17-20+ years) due to the longevity of macrofilarial infections (10–15 years). This presents a challenge in sustainability with high population coverage in mass drug administration programmes (MDA) to maintain control of disease and eventually break the transmission cycle. Further, the emergence of sub-optimal responders to IVM following repetitive treatments [5] and the risk of severe adverse reactions in patients co-infected with the bloodborne filaria, *Loa loa* [6], exert additional challenges on maintaining control of onchocerciasis.

Drugs effective at killing adult *Onchocerca* (macrofilaricides), would be desirable to address this pressing global health problem and accelerate the ultimate elimination of onchocerciasis. New macrofilaricides may well also have useful indications against the causative agents of lymphatic filariasis; LF (*Wuchereria bancrofti, Brugia malayi, B. timori*), especially to reduce the long tail of ‘endgame mop-up’ in countries that have completed extensive elimination programmes or in ‘hard to reach’ areas [1].

Existing *Onchocerca* macrofilaricides have either too low selective toxicity (suramin) [7], are known to induce severe adverse reactions contraindicating usage at doses required to be effective (e.g. diethylcarbamazine or high dose ivermectin) [8,9] or presently cannot be delivered orally (e.g. flubendazole) [10]. Whilst targeting the *O. volvulus* endosymbiont, *Wolbachia*, with tetracycline antibiotics has proven safe in delivering macrofilaricidal activity, including in individuals co-infected with *L. loa* [11,12], present protracted treatment lengths (4–6 weeks) and contraindications in certain population groups (pregnant women and children <8 years old) precludes the wide-scale use of this class of anti-*Wolbachia* drug in large scale MDA [1].

Stimulated by renewed philanthropic investment by a number of stakeholders, a number of drug discovery and development programmes have been initiated to identify new candidate macrofilaricidal therapeutics that can effectively and safely target the macrofilarial stage of *O. volvulus* or its endosymbiont in a timeframe compatible with MDA delivery (generally considered an orally administered course not exceeding seven days).

The development of *Onchocerca* macrofilaricides is hindered by the lack of an appropriate small animal laboratory model to robustly evaluate candidates *in vivo* at the pre-clinical stage. A long term model of brugian lymphatic filariasis (*B. malayi/B. pahangi*) is available in the susceptible gerbil host *Meriones unguiculatus* [13] and certain strains of inbred mice are susceptible to the filaria, *Litomosoides sigmodontis* [14]. Whilst these rodent filariasis models are undoubtedly useful in identifying efficacious filaricidal compounds, overt differences in the biology of *Onchocerca* species requires confirmation of effectiveness at the pre-clinical stage before informed decisions can be made about clinical development for onchocerciasis indications. A current example of this has been the lack of translation of macrofilaricidal activity of the macrocyclic lactone, moxidectin, from pre-clinical testing against *Brugia* in gerbils and dogs, when evaluated by a phase II clinical trial in onchocerciasis patients [15,16]. Traditionally, the use of cattle naturally infected with bovine *Onchocerca* (*O. dukei, O. gutterosa, O. ochengi, O. lienalis*) has been exploited as a pre-clinical system to scrutinise *Onchocerca-*specific macrofilaricidal activity. Throughput is severely restricted by the logistics of identifying and experimenting on parasitized cattle in sub-Saharan Africa and the large quantities of drug required to dose up to 0.5 metric tonne animals. Further, without challenging empirical pharmacological evaluation, requiring robust sample sizes, atypical pharmacokinetic profiles within ruminants may lead to erroneous conclusions regarding efficacy.

A potential solution to the current challenges facing pre-clinical onchocerciasis macrofilaricide evaluation is the development of a small animal model of macrofilarial onchocerciasis. Presently, only larval *Onchocerca* life cycle stages are routinely used to screen filaricidal drug candidates in mice. Infectious stage (L3) larvae of *O. volvulus* can be implanted within micro-chambers to achieve only abbreviated development to the L4 larval stage [17]. Microfilarial infections of the skin, utilising purified cattle *Onchocerca* mf*,* can be established in inbred laboratory mouse strains and have been utilised in pre-clinical filaricidal testing [18]. However, no reliable macrofilarial *Onchocerca* small animal model has been described. Previous attempts to utilize inbred mouse strains, including lymphopenic strains, to establish *Onchocerca* macrofilarial infections from infectious stage (L3) larval inoculations, has thus far proven unsuccessful [19]. However, Rajan and colleagues demonstrated that *O. volvulus* female worm ‘loops’, exposed from surrounding nodular encasement in excised nodules, could remain viable following implantation into Severe-Combined ImmunoDeficiency (SCID) mice [20]. The same laboratory has also demonstrated that full development of *Brugia* can occur in SCID strains [21]. Further, our laboratory has previously demonstrated that *O. lienalis* mf infections can persist for at least 100 days in SCID recipients without waning [22].

Thus, in this paper, we evaluated the suitability of the SCID mouse as a pre-clinical model to test macroflaricidal activity against filariae, using laboratory maintained *Brugia malayi* to assess responsiveness to a range of filaricidal compounds before trialling survival and drug responses against *Onchocerca ochengi* implants*.*

## Methods

### Animals

Male BALB/c SCID were purchased from Harlan Laboratories, UK, while male CB.17 SCID mice and BALB/c WT mice were purchased from Charles River, UK. Male *Meriones unguiculatus* (Mongolian gerbils; jirds) were purchased from either Charles River, UK or Janvier Laboratories, France. Rodents shipped to REFOTDE, Buea, Cameroon, were maintained in conventional housing with daily cage cleaning and changing of food. Food, water and bedding were sterilised by autoclaving. For *B. malayi* experiments, animals were kept at the Biomedical Services Unit (BSU), University of Liverpool, UK in specific pathogen-free (SPF) conditions. All experiments carried out in Cameroon were approved by the Animal Care Committee, REFOTDE. All experiments on animals in the UK were approved by the ethical committees of the University of Liverpool and LSTM, and were conducted according to Home Office (UK) requirements.

### *Brugia malayi* parasites

The life cycle of *B. malayi* (*Bm*) was maintained in mosquitoes and susceptible *Meriones* gerbils at LSTM. To generate infective *Bm* larvae (*Bm*L3) for infections, female adult *Aedes aegypti* mosquitoes were fed with *Bm* microfilariae (mf) collected from infected gerbils by catheterisation, as previously described [23], followed by mixing with human blood and feeding through an artificial membrane feeder (Hemotek**®**). Blood-fed mosquitoes were reared for 14 days to allow for development to L3. The L3 were collected from infected mosquitoes by crushing and concentration using a Baermann’s apparatus and RPMI medium.

### *Onchocerca ochengi* parasites

*O. ochengi* nodules were obtained from the skins of naturally infected zebu cattle from the Adamawa region of Cameroon through existing commercial practice for meat production. At abbatoirs in the South West Province, Cameroon, female cattle were checked for *O. ochengi* infection by palpation of the skin of the umbilical region for onchocercomata. Positive skins were collected after slaughter. The tissue was then transported to REFOTDE, Buea within 2 hours of collection. Skins were washed several times and the hair was removed by shaving. Onchocercomata were excised from dermal tissue using sterile scalpels and forceps, and were placed in RPMI containing penicillin, streptomycin and neomycin (RPMI + PSN). To obtain free adult males, onchocercomata were cut gently to expose the adult worms using sterile scalpels and were incubated in petri dishes containing RPMI + PSN for 4 hours at 37°C, 5% CO_2,_ to allow males to escape into the medium. Free, intact and motile adult males were confirmed by visualisation of posterior anatomy, were washed several times in fresh RPMI + PSN and kept at 37°C, 5% CO_2_ overnight.

### *B. malayi* experimental infections

For *B. malayi* (*Bm*) L3 infection, 100 freshly collected, motile *Bm* L3 were injected via the intra-peritoneal route. Efficiencies of inoculations were confirmed by needle washout. For mf perfusion, *Bm* mf were harvested by peritoneal washings of patently infected *Meriones* gerbils under isoflurane anaesthesia, washed in RMPI + PSN and purified by PD10 column size exclusion chromatography (Amersham). *Bm* mf were enumerated by microscopy, concentrated by centrifugation and 0.125 × 10^6^ mf were inoculated into the circulation via the lateral tail vein as described previously [24].

### Implantation of adult macrofilariae

*Bm* macrofilariae were collected from infected SCID mice and were grouped into batches of 13 adults. Male BALB/c SCID and BALB/c WT mice were placed under surgical anaesthesia using isofluorane and were given s.c. injection of bruprenorphine before 13 *Bm* macrofilariae were placed into the peritoneal cavity by making a small incision to the skin and abdominal cavity wall in the upper right quadrant. The incisions were re-sutured after implant and animals were individually housed after surgery.

For *Onchocerca* implants, rodents were placed under surgical anaesthesia using i.p. injections of ketamine and medetomidine. Onchocercomata were thoroughly cleaned of bovine tissue, washed in several changes of RPMI + PSN and small sections of the adult worms were exposed by partial rupture of the capsule. Groups of 8–15 motile *O. ochengi* male macrofilariae or 4 prepared *O. ochengi* nodules were implanted into the peritoneal cavity or to the cutaneous tissue of the upper side of the neck (for s.c nodule implants). All wounds were re-sutured after surgery and animals were individually housed and closely monitored for the recovery period (7 days post-op).

### Drug treatments

The drug doses in mg/kg and routes used in this study were: Albendazole (ABZ; 50 mg/kg qd po), diethylcarbamazine (DEC; 50 mg/kg qd po), flubendazole (FBZ; 10 mg/kg qd sc), ivermectin (IVM; 5 mg/kg qd ip or 15 mg/kg qd po), minocycline (MIN; 25 mg/kg bid po) and rifapentine (RIFAP; 15 mg/kg qd po). Drugs were dissolved in standard suspension vehicle (0.5% sodium carboxymethyl cellulose; 0.5% benzyl alcohol; 0.4% Tween 80; 0.9% NaCl), with the exception of MIN, which was dissolved in water, RIFAP which was dissolved in 55% polyethylene glycol 300; 25% propylene glycol; 20% water and parenteral IVM which was dissolved in 1% DMSO. Oral drugs were administered in volumes of 100-200 μl by gavage. All drugs were purchased from Sigma Aldrich.

### Drug efficacy assessments

Developing larvae, macrofilariae and released mf were recovered by peritoneal washings and enumerated by microscopy. Motile, unencapsulated worms were scored as viable. *Bm* mf were centrifuged before being resuspended in a known volume and a sample enumerated. For *Bm* mf microfilaraemias, 30 μl thick films on uncoated glass slides (Corning) were prepared from freshly-collected blood (tail bleeds or cardiac punctures post-mortem) in 5000 U/ml lithium heparin anti-coagulant (Sigma Aldrich). Slides were air dried, de-haemaglobinised in tap water, fixed in 80% MeOH and stained with Giemsa (BDH). *Bm* mf were enumerated by microscopy. For recovery of *O. ochengi* nodules, the nodule tissue was excised from the area of tissue engraftment post-mortem. Nodules were dissected and incubated in RPMI + PSN, 37°C, 5% CO_2_ for +4 h before the presence of motile worms or mf was evaluated by microscopy.

### PCR quantification of *Wolbachia*

Individual adult filariae were fixed in RNAlater and stored at 4°C. For *Wolbachia* enumerations, DNA was extracted from worm samples using the DNeasy Blood and Tissue Kit (Qiagen) according to manufacturer’s instructions. Levels of *Bm Wolbachia wsp* and *Bm gst* gene copy numbers were quantified using qPCR with *Bm-*specific primers, as reported elsewhere [25]. Levels of *O. ochengi-*specific *wsp* and *gst* were estimated using identical PCR conditions, with the *wsp* primer pairs: *wsp*420TGTTGGT(AG)TTGGT(GC)TTGGTG, *wsp*583AACCAAA(AG)TAGCGAGC(CT)CCA and the *gst* primer pairs: *gst*175ATTGAAGCGCTTATTAGTCTGC, *gst*305TGTCGTTTCCATTTCATTTTC.

### Statistical analysis

Where intra-group data was skewed, non-parametric analyses were used to compare statistical differences between two independent groups (Mann Whitney test) or three or more independent groups (Kruskal Wallis with Dunn’s post-hoc tests). Where intra-group data displayed a normal distribution, parametric 1way ANOVA with Bonferroni post-hoc tests were used to examine statistical differences between 3 or more groups. Paired T tests evaluated changes in time within individual animals. Correlations were assessed for significance using Pearson Correlation test. Significance is indicated by P < 0.05*, P < 0.01** and P < 0.001***.

## Results

### Evaluating the performance of reference filaricidal compounds against *B. malayi* in SCID mice

We adapted mouse models of Brugian filariasis [26-28] to scrutinise whether the standard anti-filarial drugs (SAFD) albendazole (ABZ), diethylcarbamazine (DEC) and ivermectin (IVM) or drugs with demonstrated macrofilaricidal properties, flubendazole (FBZ) and the tetracycline anti-*wolbachia* antibiotic, minocycline (MIN), displayed predictable efficacy in a Severe-Combined Immuno-Deficiency (SCID) mouse model system.

**Figure 1:**
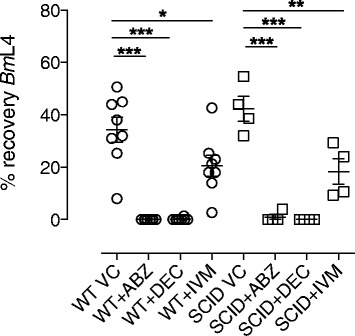
**Comparative larvicidal activity of SAFDs against** ***Brugia*** **in WT versus SCID mice.** Percentage recoveries of *Bm*L4 larvae from the peritoneal cavities of mice eight days after inoculation with 100 *Bm*L3 and commencement of a seven day oral regimen of 50 mg/kg qd albendazole (ABZ), diethylcarbamazine (DEC) or 15 mg/kg qd ivermectin (IVM). Circle plots are recoveries from BALB/c WT mice whilst square plots are derived from BALB/c SCID mice. Error bars are mean +/−SEM% recoveries per group. Data is pooled from two individual experiments for WT and one experiment for SCID mice (n = 4/group). Significant differences (1 way ANOVA with Bonferroni multiple comparison) are indicated P < 0.001***, P < 0.01**, P < 0.05*.

For SAFD, which predominantly target the larval stages of filariae in clinical and veterinary indications, we inoculated either BALB/c SCID or BALB/c wild type (WT) mice, as an immune-sufficient control, with 100 infectious third stage (L3) of *Brugia malayi* (*Bm*) via the intra-peritoneal route. Infected mice were orally administered with SAFD or vehicle control (VC) at indicated doses for seven days, before *Bm* larvae were recovered by peritoneal lavage (+24 h following the final drug exposure) and motile parasites enumerated. Results are shown in Figure 1. ABZ and DEC treatment delivered an almost complete filaricidal activity in WT mice (100% and >99%, respectively). These potent filaricidal efficacies were emulated within SCID hosts (ABZ; 98%, DEC; 100% reductions, *P < 0.0001*). IVM was partially filaricidal at the dose administered (15 mk/kg qd) in WT mice (39.8% mean reduction, *P = 0.0356*). In SCID mice, a partial filaricidal response was also evident (56.7% mean reduction, *P = 0.026*).

**Figure 2:**
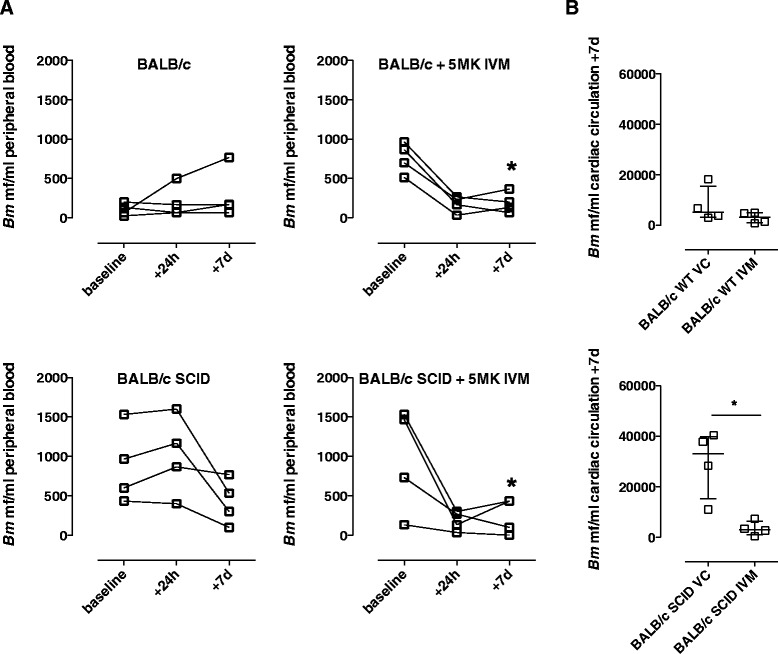
**Comparative microfilaricidal activity of IVM against** ***Brugia*** **in WT versus SCID mice.** *Bm* mf densities in peripheral circulating blood **(A)** or cardiac circulation **(B)** at ‘baseline’ (48 h following intravenous perfusion with 1.25 × 10^5^ purified mf) or at indicated time points following treatment with 5 mg/kg ivermectin (IVM) or vehicle control (VC) via the intraperitoneal route. Top panels are data derived from WT BALB/c, bottom panels are data from BALB/c SCID mice. Error bars indicate median mf densities and interquartile range. Data is from an individual experiment (n = 4/group). Significant differences with time (paired T Test) or between groups (Mann Whitney Test) are indicated *P < 0.05.

To further assess responses of the SAFD, IVM, against mf in the circulation, *Bm* mf were inoculated via the tail vein into BALB/c SCID mice or BALB/c immune competent controls. Initial parasitaemias were measured at a baseline of +48 h after inoculation, before groups of mice were administered with a single parenteral dose of 5MK IVM or VC. IVM induced rapid reductions in microfilaraemia +24 h post-treatment and levels further declined +7d post-treatment in both WT and SCID mice (79%; *P = 0.0194* and 76%; *P = 0.0473* median reductions +7d, respectively, Figure 2A). In SCID mice, the levels of mf recovered from the cardiac puncture at termination were also significantly reduced in IVM-treated mice (89% median reduction, *P = 0.0286,* Figure 2B).

**Figure 3:**
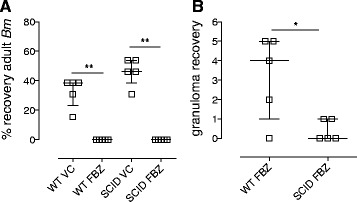
**Comparative macrofilaricidal activity of FBZ against** ***Brugia*** **in WT versus SCID mice. (A) **% recoveries of *Bm* macrofilariae and **(B)** numbers of granulomas recovered +6 weeks after intraperitoneal surgical implantation into BALB/c WT or BALB/c SCID mice and commencement of a 5 day 10 mg/kg qd sub-cutaneous dosing with flubendazole (FBZ) or matching vehicle control (VC). Error bars indicate median and interquartile range. Data is from an individual experiment (n = 5/group). Significant differences between groups (Mann Whitney Test) are indicated *P < 0.05, **P < 0.01.

Whilst infectious stage *Bm* L3 are reported to develop into mf-producing macrofilariae in SCID mice, WT mice are refractory to full *Bm* development [21,26]. For the purposes of validating direct FBZ macrofilaricidal efficacy in immunodeficient mice compared with immunosufficient controls, we implanted a defined number of *Bm* macrofilariae sourced from +35 day ip infections of SCID donors into either WT or SCID recipients. Following +2 day recovery from surgery, mice were placed on parenteral FBZ suspension (10 mk/kg sub-cutaneous, qd) or matching VC for a total of five days and macrofilaricidal efficacy was evaluated +5 weeks following the last drug exposure. Comparing VC groups, there was a marginally non-significant decreased recovery of *Bm* macrofilariae in BALB/c WT versus BALB/c SCID mice (*P = 0.069*). FBZ treatments were 100% efficacious in both WT and SCID BALB/c mice implanted with *Bm* macrofilariae (Figure 3A; average recovery of 0% FBZ treatment *vs*. 38%, *P = 0.0067* for VC WT and 46% *P = 0.0071* for VC SCID). Entrapped immotile macrofilariae encapsulated within leukocytic granulomas were frequently evident in WT but not SCID mice (median recovery 4, WT *vs* 0, SCID, *P = 0.0104*; Figure 3B).

**Figure 4:**
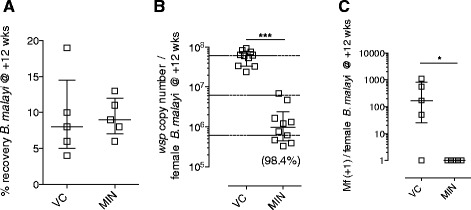
**Anti-** ***Wolbachia*** **activity against** ***Brugia*** **macrofilariae in SCID mice. (A) **% recoveries of *Bm* macrofilariae **(B)** *Wolbachia* loads per female *Bm* and **(C)** numbers of released mf per female *Bm* in BALB/c SCID mice +12 weeks after intraperitoneal infection with 100 *Bm*L3 and +6 weeks after commencement of 4 week oral 25 mg/kg bid minocycline (MIN) or vehicle control (VC). Error bars indicate median and interquartile range. Dashed horizontal lines in (B) indicate 1 and 2 log reductions compared with median VC levels. Median % reduction in **(B)** is indicated in parentheses. Data is from an individual experiment (n = 5/group; individual worms pooled per group). Significant differences between groups (Mann Whitney Test) are indicated *P < 0.05, ***P < 0.001.

We took advantage of the full development of *Bm* in SCID mice to examine the response to oral treatment with the anti-*Wolbachia* tetracycline, MIN, against the macrofilarial stage of the parasite. SCID BALB/c mice were infected with 100 infectious stage *Bm*L3 larvae ip and dosing with MIN or vehicle control (VC) commenced at +6wk post-infection, when parasites had reached the juvenile adult stage. Mice were left for two weeks following the last drug exposure and adult *Bm* and released mf were collected at the early onset of patency (+12wks post-infection). The average recovery of adult *Bm* in control mice was 9.4 +/−2.6 SEM (n = 5) and recoveries did not vary in MIN treated animals (Figure 4A). Numbers of *Wolbachia* were quantified following DNA extraction of individual female worms (n = 10/group, pooled from individual mice; Figure 4B). In the control group, average *Wolbachia* numbers per female were 5.865 × 10^7^ +/−0.72 × 10^7^. The average number of *Wolbachia* in female worms exposed to MIN *in vivo* in SCID mice had been significantly reduced to 1.837 × 10^6^ +/−0.702 × 10^6^. This equated to a 98.4% reduction in *Wolbachia* (*P < 0.0001*). The majority of control mice also contained peritoneal mf (4/5 mice; Figure 4C). Mf production was enumerated per female worm and, on average, each 12 week old female worm had produced 373.3 (+/−204.4) mf. Contrastingly, there was a complete absence of mf in the peritoneal cavity of MIN treated SCID mice *(P = 0.0215)*, consistent with loss of embryonic development following the sterilisation of *Wolbachia* from adult *Bm* filarial tissues.

### Yields of *Onchocerca* macrofilariae from naturally parasitized cattle

**Figure 5:**
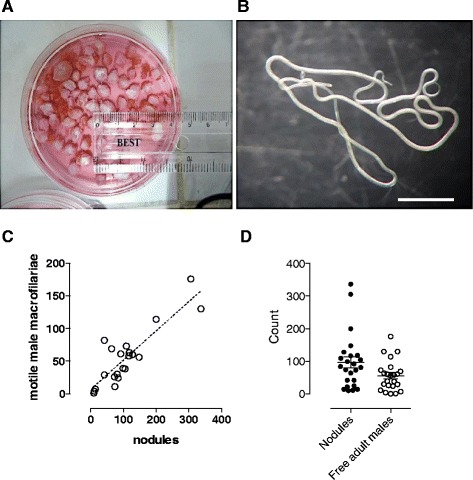
**Yields of female and male** ***Onchocerca*** **macrofilariae isolated from naturally infected cattle. A)** Typical yield of excised *O. ochengi* onchocercomata from a parasitized cattle hide **B)** A pair of liberated, motile male *O. ochengi* worms *+*4 h after disruption of onchocercoma (nodule) capsule and culture at 37°C/5%CO_2_. Scale bar =1 cm. **C)** Relationship between numbers of onchocercomata recovered per cattle hide and number of motile male *O. ochengi* harvested. Dashed line is linear regression best fit. **D)** Recoveries of onchocercomata (closed plots) and male *O. ochengi* (open plots) per cattle hide (n = 29). Error bars are mean +/− SEM.

Zebu cattle, naturally infected with the bovine *Onchocerca* species, *O. ochengi*, were identified within herds moved from pasture land in the Adamawa Region and slaughtered for meat production in abattoirs located in the South West region of Cameroon. Over a 48-day collection period, a total of 2612 nodules were collected from 28 infected hides (mean 105.5 +/− 28.4 SEM, Figure 5), which were processed to purify motile male *Onchocerca* macrofilariae. A positive correlation between numbers of nodules recovered and numbers of adult males was observed (Pearson R = 0.869; *P < 0.0001*) and, on average, 69.7 +/− 16.4 SEM motile male macrofilariae were recovered per infected cattle hide processed. This was equivalent to an average ratio of 0.66:1 male macrofilariae recovered per harvested nodule.

### *Onchocerca* macrofilariae survival following implantation into WT/SCID mice and Mongolian gerbils

**Figure 6:**
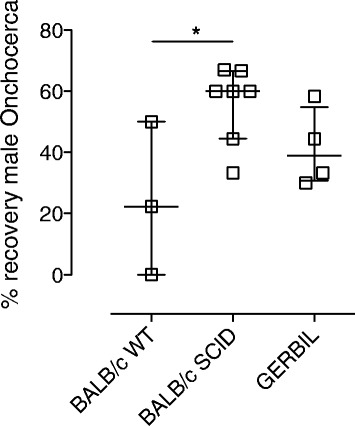
**Survival of male** ***Onchocerca*** **macrofilariae in laboratory rodents.** Percentage recoveries +15 days post-intraperitoneal implantation with 8–12 isolated, motile male *Onchocerca* macrofilariae in BALB/c WT, BALB/c SCID and *Meriones unguiculatus* gerbils. Error bars are median recoveries with interquartile range. For BALB/c SCID, data is pooled from two individual experiments (n = 3/4). For BALB/c WT and *Meriones,* data is from a single experiment (n = 3/4). Significant differences between groups (Kruskal-Wallis Test with Dunn’s multiple comparison) is indicated *P < 0.05.

Using the intraperitoneal surgical implant technique as detailed above for *Bm*, we implanted isolated motile male *Onchocerca* macrofilariae into various laboratory rodent models and evaluated survival following +15 days (Figure 6). As well as comparison of WT vs SCID mice, we tested survival in Mongolian gerbils (*Meriones unguiculatus*), an outbred laboratory rodent susceptible to a number of other filariae. After 15 days post-surgical implant, we successfully recovered parasites from all rodent recipients with the exception of one WT BALB/c mouse. Male *Onchocerca* macrofilariae recovered from rodent implants were monitored for viability *ex vivo* in culture after recovery from rodent hosts and motility comparable to freshly isolated adult males was observable up to 7 days. At +15 days, recoveries of male macrofilariae were, on average, significantly lower in WT BALB/c versus SCID BALB/c mice (22% *vs* 60% median recovery, *P = 0.0475*), whilst the average recovery of *Onchocerca* male macrofilariae was not significantly different in *Meriones* gerbils *vs* WT or SCID BALB/c mice (38%).

**Figure 7:**
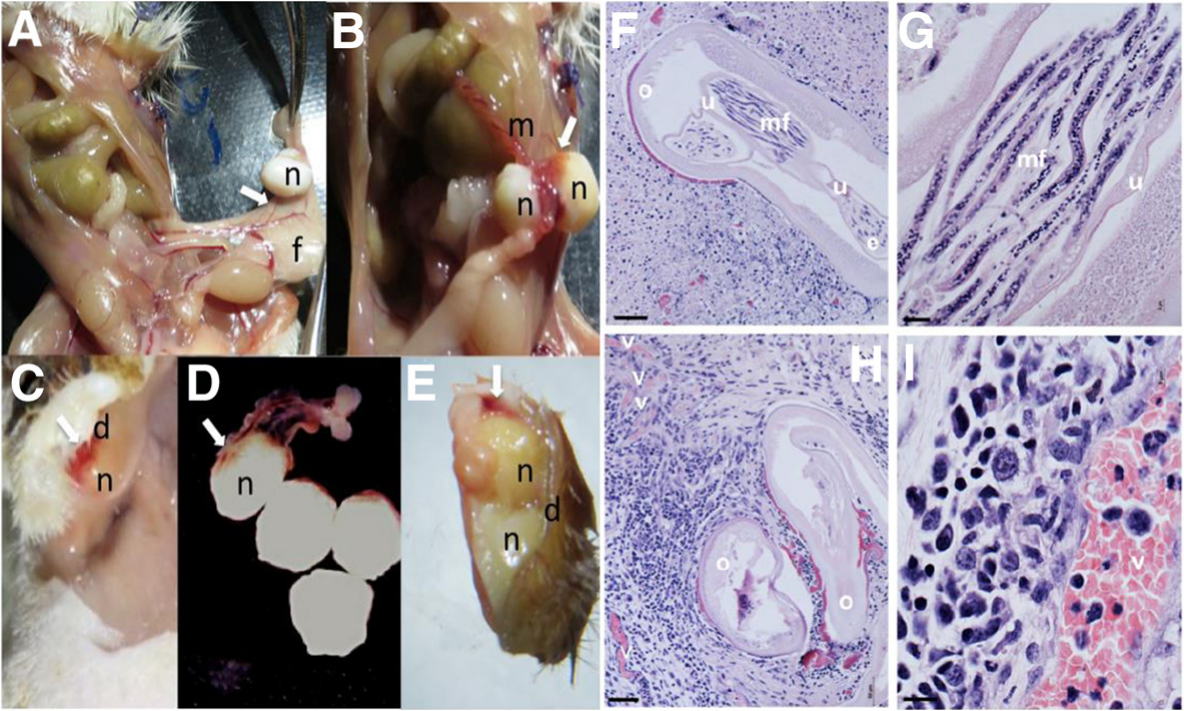
**Engraftment of** ***Onchocerca*** **onchocercomata in SCID mice.** Engrafted peritoneal **(A&B)** or sub cutaneous **(C&D)** onchocercomata *in situ* or excised nodules from the peritoneum **(D)** or sub cutaneous tissue **(E)** +7 days after implantation into BALB/c or CB.17 SCID mice. Haematoxylin and eosin staining of sub cutaneous engrafted onchocercomata illustrating embryogenesis **(F)** and putative murine host vessel-like structures **(H)**. High magnification images of inter-uterine stretched microfilarae **(G)** and vessel-like structure **(I)**. Key: dermis (d), embryos (e), visceral fat (f), mesentery (m), microfilariae (mf), nodule (n), female *Onchocerca ochengi* (o), uteris (u), vessel (v). Arrows indicate zones of neovascularisation. Scale bars are 50 μm (F&H) and 10 μm (G&I).

We also trialled the implantation of isolated *O. ochengi* onchocercomata, containing a mixture of cow tissue plus both female and male *Onchocerca* macrofilariae, into SCID BALB/c or SCID CB.17 strains. In this pilot study, four onchocercomata were implanted into the peritoneal cavity or sub-cutaneously at the nape of the neck of each SCID recipient. Table 1 and Figure 7 detail the parasitological observations +7 days following nodule implantation. At necropsy, the majority of SCID BALB/c recipients (4/6) had evidence of engraftment of some or all implanted onchocercomata into abdominal visceral tissues, mainly connective tissues continuous with the mesenteries and visceral fat pads. In engrafted onchocercomata, neovacularisation could be observed (9/24 implanted nodules), manifest as capillary beds distributed across the capsule surface, stemming from proximal host vascular networks. Onchocercomata were extracted, partially dissected to expose loops of female macrofilariae and cultured *ex vivo* for 4 hours. Observations of motility during this period revealed that in 5/6 SCID recipients, 100% of implanted nodules contained motile female *Onchocerca* macrofilariae. Transplanted onchocercomata yielded motile male *Onchocerca* macrofilariae after recovery from 4/6 SCID recipients at a ratio similar to that observed from freshly isolated nodules (i.e. ~1 male/2 nodules). Following sub cutaneous implantations in SCID CB.17 mice, 100% of onchocercomata (n = 8) in 2/2 recipients displayed engraftment into the sub cutaneous layer with evidence of neovascularisation. Vessel-like structures containing blood cells, proximal to female worms, were identifiable within implanted onchocercomata by histology. All onchocercomata recovered from sub cutaneous implants at +7 days contained motile female macrofilariae when dissected and cultured *ex vivo.* A single motile male macrofilaria was released from nodules derived from 1/2 recipient mice. Evidence of embryogenesis within implanted female *Onchocerca* uteri was apparent by histological examination, including the presence of inter-uterine stretched mf. Further, released motile microfilariae were evident in the culture medium following *ex vivo* culture of implanted sub cutaneous onchocercomata derived from 2/2 SCID CB.17 recipients.

**Table 1:** **Parasitological observations of** ***Onchocerca ochengi*** **onchocercomata post-implantation into SCID mice**

**Mouse strain/ID**	**Number/location**	**Vascularisation**	Motile females*	Motile males*	Motile mf*
BALB/c SCID 1	4 ip	4/4	4/4	2/4	nd
BALB/c SCID 2	4 ip	2/4	4/4	0/4	nd
BALB/c SCID 3	4 ip	0/4	4/4	0/4	nd
BALB/c SCID 4	4 ip	0/4	4/4	2/4	nd
BALB/c SCID 5	4 ip	1/4	0/4	2/4	nd
BALB/c SCID 6	4 ip	2/4	4/4	2/4	nd
CB.17 SCID 1	4 sc	4/4	2/2	0/2	yes
CB.17 SCID 2	4 sc	4/4	3/3	1/3	yes

Intraperitoneal implantation (ip), sub cutaneous implantation (sc).

*motility assessed by dissection microscope +4 h after excision of nodule grafts, disruption of nodule capsule and culture in complete medium at 37°C/5%CO_2_.

nd – not done.

### Evaluation of the SCID model of onchocerciasis as a pre-clinical macrofilaricidal drug screen

**Figure 8:**
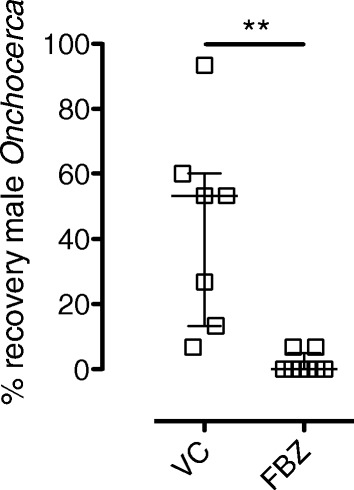
**Macrofilaricidal activity of parenteral FBZ against** ***Onchocerca*** **macrofilariae in SCID mice.** Percentage recoveries +5 weeks after intraperitoneal surgical implantation of 15 isolated, motile male *Onchocerca* macrofilariae in BALB/c SCID mice and commencement of a 5 day 10 mg/kg qd sub-cutaneous dosing with flubendazole (FBZ) or matching vehicle control (VC). Error bars indicate median and interquartile range. Data is from an individual experiment (n = 7-8/group). Significant differences between groups (Mann Whitney Test) are indicated **P < 0.01.

Because of initial, reproducible, high recoveries of viable male *Onchocerca* macrofilariae in BALB/c SCID mice, we evaluated this model as an anti-*Onchocerca in vivo* macrofilaricidal drug screen where survival over a more protracted time frame (4–5 weeks) would be necessary to evaluate macrofilaricide efficacy. We applied the optimised FBZ screen verified in BALB/c SCID *Bm* implants (Figure 3) to compare suitability of this system for macrofilaricide testing. An increased number of 15 motile male *Onchocerca* macrofilariae were implanted into each SCID mouse recipient to mitigate against decline in survival over 5 weeks. Also, daily isolates of macrofilariae (from individual cattle hides) were divided equally between recipients consigned to FBZ or VC treatments to avoid inter-group bias in quality and age of male worms that might affect survival. Further, to mitigate against inter-group variation in survival rates over a protracted period post-implantation masking macrofilaricidal effects, group sizes were increased to 7–8 mice. Treatment groups were maintained for up to 31 days (4–5 wks post implant) before recovery of parasites (Figure 8). FBZ induced a significant, almost total macrofilaricidal response, with mean survival of 1.67% (+/−1.09, n = 8), compared with a mean survival of 43.81% (+/−11.44, n = 7) recovery in the VC group (*P =* 0.0089). In the treated group, single motile worms were recovered in 2/8 recipients, whilst the other six mice had either an absence of infection or recovery of completely immotile worms. The two motile worms derived from FBZ treated mice were moribund with irregular and retarded ‘twitching’ motility, compared to the motility rate observed of macrofilariae recovered from VC mice or of freshly isolated onchocercomata.

**Figure 9:**
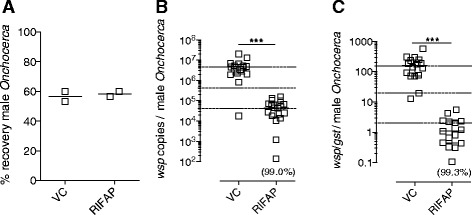
**Anti-** ***Wolbachia*** **activity against** ***Onchocerca*** **macrofilariae in SCID mice. (A)**% recoveries, **(B)** *Wolbachia* loads per motile male *Onchocerca* macrofilaria measured by *wsp* gene copy number or **(C)** *Wolbachia* loads per motile male *Onchocerca* macrofilaria as a ratio *wsp/gst* filarial gene copy number +29 days after intraperitoneal implantation in CB.17 SCID mice and +28 days after commencement of 2 week oral 15 mg/kg rifapentine (RIFAP) or vehicle control (VC). Error bars indicate median and interquartile range. Dashed horizontal lines indicate 1 and 2 log reductions compared with median VC levels. Median % reduction is indicated in parentheses. For B&C, data from individual worms are pooled from groups of two mice. Significant differences between groups (Mann Whitney Test) are indicated ***P < 0.001.

We also tested a lead anti-*Wolbachia* rifamycin antibiotic, rifapentine (RIFAP), for activity against *Onchocerca* male worms in CB.17 SCID mice. Groups of two mice were given oral doses of RIFAP (15 mg/kg) or standard suspension vehicle (VC) daily for 14 days, starting from 1–2 days recovery after surgery. Implanted *Onchocerca* were retrieved +2 weeks after last drug exposure. Total recoveries of *Onchocerca* macrofilariae were not affected by treatment regimen (56.65%, VC and 58.35%, median recovery, RIFAP; Figure 9A). Numbers of the single copy *Wolbachia* surface protein (*wsp*) gene per isolated male *Onchocerca* marofilariae were quantified as a surrogate measurement of endosymbiont density within nematode tissues (Figure 9B). A highly significant 99% reduction in *Wolbachia* number was recorded in *Onchocerca* macrofilariae obtained from CB.17 SCID mice treated with rifapentine for 14 days (4.6 × 10^6^ median *wsp* copies, VC *vs.* 0.0452 × 10^6^, RIFAP, *P < 0.001*). *Wolbachia* reductions were preserved when adjusted for potential variation in size and age of adult male *Onchocerca* derived from cattle onchocercomata, by normalisation to a single copy filarial gene, *gst* (Figure 9C).

## Discussion

The development of macrofilaricides against onchocerciasis is currently hampered by a lack of a facile pre-clinical infection model. Extrapolating efficacy of drug candidates against lymphatic filariae in susceptible rodents may not necessarily translate into effective onchocerciasis indications and traditional pre-clinical testing in cattle onchocerciasis does not possess the required throughput to address current demand. For these reasons we decided to develop and validate a small animal model of onchocerciasis. We chose to trial SCID mice as a compatible host for the cattle *Onchocerca, O. ochengi.* We selected this particular parasite after identifying an abundant and relatively convenient sampling source of *O. ochengi* macrofilariae in cattle herds derived from the Adamawa region of Northern Cameroon used for commercial meat production. Our experience over a three-month evaluation period indicated that the prevalence of infected female cattle being moved for slaughter in the South West Province was typically between 5-10% and that, with around 10–20 cattle being processed daily at a local abattoir, there was frequent availability of infected cow tissues. Because we typically collected >100 *O. ochengi* macrofilariae from a single hide, this provided an adequate daily supply line of *Onchocerca* for *in vivo* drug testing. The implantation of macrofilariae into groups of rodents from a single cow effectively increased capacity 4–5 fold in terms of biological units available for drug testing. Further, considering the much reduced space and costs demanded for long-term husbandry of rodents *vs* cattle, the rodent model should further facilitate increased capacity for simultaneous or overlapping *Onchocerca* drug screening within the same laboratory, in a cost-effective manner. The ability to culture the male *O. ochengi* macrofilariae *ex vivo* for durations of at least five days, also means that this method of collection could be applied to *in vitro* testing of novel macrofilaricides, similar to the published male *O. gutterosa* drug screen [29].

The selection of SCID mice was based on extensive use of this lymphopenic mouse system for xenograft transfers in cancer and malaria chemotherapy pre-clinical testing [30,31] and our own and other laboratory’s observations that SCID mice are permissive hosts for non-murine filariae, including full development of the human lymphatic filariae, *B. malayi* [21] and stage-specific *Onchocerca spp.* infections [20,22].

A potential caveat to the application of SCID mice for anti-filarial drug screening is the absence of adaptive immune responses, which might be important in interacting with candidate filaricidal compounds to induce macrofilaricidal effects. Whilst it is inconclusive whether inflammatory reactions in patients post-treatment with anti-filarial drugs (e.g. “Mazzoti reactions”) are a necessary component in the death of filariae or merely a response to dead and damaged worms [16] and the concomitant liberation of somatic antigens and *Wolbachia* endobacteria [32,33], the former has been proposed due to a general lack of efficacy of SAFD at physiological levels *in vitro* against filariae [34,35]*.* Further, various ‘immuno-pharmacological’ modes of action have been proposed for SAFD, including the prevention of immuno-modulatory secretions from mf by IVM [36] and the induction of host inducible nitric oxide and cyclooxygenase pathways by DEC [24]. Thus, it was important to validate anti-filarial responses in the SCID model against an immunologically intact WT control, where possible. For this we took advantage of WT BALB/c mice as an immune competent background strain that, whilst resistant to chronic patent infections, accommodates life cycle stages of *B. malayi* for periods sufficient to evaluate filaricidal drug effects [24,27]. The results of our studies, comparing larvicidal responses of the SAFD: ABZ, DEC and IVM, illustrated no difference in the level of observed efficacy of these reference anti-filarials against infectious *Bm* L3 larvae, or in the case of IVM, bloodborne mf, in the absence of adaptive immune responses. In fact, for mf, we observed a 2.5 fold higher peripheral circulating mf and 6.4 fold increased cardiac blood levels in SCID vs WT control groups at 9 days post-tail vein perfusion, which allowed us to discern a more obvious treatment effect of single dose IVM. We also observed similar mf levels in CB.17 SCID mice (data not shown). The elevated persistence of circulating mf in SCID mice may be due to a lack of initiation of an effective stage-specific adaptive immune response. Certainly, microfilariaemic brugian filariasis patients have hyporesponsive filarial antigen-specific peripheral blood mononuclear cell responses vs amicrofilaraemic infected individuals [37]. Splenic clearance has been demonstrable in controlling bloodborne microfilaraemias in mice [38] and baboons [39] which suggests that SCID mice may have a defective mechanism at this secondary lymphoid organ.

This data extends experiments undertaken in athymic nude mice that demonstrates a lack of a role for T lymphocytes/lymphocyte ‘help’ in a DEC mode of action [40] and also suggests T and B lymphocyte-mediated adaptive immunity is dispensable for the effects of the benzimidazole (BZ) and macrocyclic lactone families of anti-filarial drugs. Further, a complete FBZ macrofilaricidal response was apparent in both WT and SCID animals using a dose regimen previously reported as effective in the *Meriones* gerbil *Brugia* macrofilariae implant model [41]. FBZ macrofilaricidal efficacy generally proceeded without overt leukocytic granuloma formation in SCID mice suggesting that these reactions are a response to dead worms but are not integral to the mode of action of BZ anthelmintics. Whether this extends to other classes of SAFD that have macrofilaricidal activity against *Bm*, e.g. DEC and MOX, remain to be evaluated.

We extended our evaluation of the SCID mouse, taking advantage of the full development of *Brugia* in this immunocompromised system, to assess the appropriateness of its use as a facile anti-*Wolbachia* drug screen. For this we chose a point of oral administration to drug with the anti-*Wolbachia* reference tetracycline, MIN, once parasites had undergone the final moult to become immature macrofilariae. At this point in the *Bm* life cycle, *Wolbachia* numbers have completed a log phase expansion and are representative of levels in mature *Brugia* (1-5 × 10^7^*Wolbachia* per female worm) [25]. Further, initiating anti-*Wolbachia* drugging at the immature macrofilarial stage allowed us to simultaneously and rapidly discern downstream effects on embryogenesis via initial mf release, at the immediate onset of patency (+10-11 weeks in mice). We could consistently recover *Bm* macrofilariae in infected and dosed SCID animals, supporting prior observations of full permissiveness of this model [21] and allowing for reliable production of the required numbers of adult worms and mf release for assessment of *Wolbachia* efficacy. Oral MIN administration in infected SCID mice for 28 days reduced *Wolbachia* loads within female *Bm* below the 90% threshold deemed to irreversibly sterilise filarial tissues in clinical studies, leading to macrofilaricidal effects [33,42,43]. The >90% levels of depletion observed in female macrofilariae derived from drugged SCID *Bm* infections were consistent with *in vivo* effects reported following protracted tetracycline treatments in patent *Bm* infections of gerbils [44] and were reflected in a complete blockade in mf release in drugged animals.

Having validated the SCID model system as suitably responsive to a range of filaricidal reference compounds, we tested survival rates of male *Onchocerca* macrofilariae in either SCID BALB/c or WT BALB/c mice. The results of a two-week implant pilot study indicated that male *Onchocerca* survived significantly better in the immunocompromised system, suggesting that adaptive immune responses begin to exert attritional effect on survival by this stage. This is consistent with the kinetics of immune-attrition against larval *Bm* infections where adaptive immune responses begin to exert significant filaricidal activity at +2 weeks post-infection [26]. We were also able to preliminarily evaluate survival in the *Brugia* susceptible outbred rodent, *Meriones,* which indicated that male *Onchocerca* macrofilariae could persist at a similar level as within SCID mice. As our focus was on development of a flexible ‘pan-filarial’ SCID pre-clinical model, we did not investigate survival in *Meriones* gerbils further. However our initial findings suggest *Meriones* may also be a suitable laboratory host for male *Onchocerca* implants and further investigation is warranted to explore the length of persistence in this immune-intact outbred rodent. It is debatable whether macrofilaricides with a total reliance on adaptive immunity would be suitable for indications in chronic filariasis patients who typically display hyporesponsive T cell profiles to filarial antigens [37,45,46]. Furthermore, identification of ‘hits’ within *in vitro* culture screening assays [29,47] would fail to identify drugs with a reliance on host adaptive immune responses and as such, these candidates would be suitable for *in vivo* evaluation in SCID mice. However, should drugs emerge with putative T or B cell adaptive immune-pharmacological mechanisms, the availability of comparative *Bm* and *Onchocerca* macrofilaricide screens in WT mice, SCID mice and gerbils would serve as useful pre-clinical assessment tools to dissect the mode of action and spectrum of anti-filarial effects.

By applying the efficacious FBZ regimen demonstrable to exert macrofilaricidal effects against *Bm* adults, we could reproduce the profound effect of this drug against implanted adult male *Onchocerca* macrofilariae within SCID mice. This tallies with the reported macrofilaricidal effect of the modified FBZ, UMF078, against *O. ochengi* in the natural cattle host [48]. Thus, our experiment demonstrates ‘proof of principle’ that the SCID mouse *Onchocerca* macrofilariae implant model is sufficiently robust to test macrofilaricide activity over a 5 week time frame. Because single, moribund yet motile worms could be isolated in a minority of FBZ treated mice, a slightly extended period may be warranted to determine maximum macrofilaricidal activity. Male macrofilariae were reproducibly recovered from 100% of 22 SCID mouse recipients in our experiments (discounting FBZ treatments), assessed between 2–5 weeks post implant (mean survival = 49.22% +/−5.08). It is probable that more protracted durations of survival are achievable and this requires further assessment.

Confirming our validation experiments with *Bm,* the male *Onchocerca* macrofilariae SCID implant model was also assessed as a suitable anti-*Wolbachia* screen, with strong *Wolbachia* signal being reliably detected from individual male *Onchocerca* worms and >98% depletions of *Wolbachia* from filarial tissues recorded following ‘gold-standard’ oral RIFAP treatment. This is consistent with the rapid effects of the rifamycin class of antibiotics observable in the *B. malayi* infection model in SCID mice (manuscript in preparation).

The matching anatomical site of *Bm* adult parasitism and *Onchocerca* macrofilariae implantation within the peritoneal cavity of the pan-filarial SCID pre-clinical screen, along with availability of both *Brugia* and *Onchocerca* macrofilariae *ex vivo* screening systems, offers a comprehensive suite of pre-clinical tools for robust interrogation of novel macrofilaricide and anti-*Wolbachia* drugs in development. This is further aided by a high throughput cell screening assay for anti-*Wolbachia* drug discovery [49]. The pan-filarial murine SCID host described here will therefore provide a useful tool to facilitate the construction of rational PK/PD models and help drive iterative medicinal chemistry to further improve promising classes of drugs. Perhaps the most important advance this model provides is the ability to more accurately discern comparative drug class effects against different genera of medically important filariae in a controlled experimental system. Should the *Bm* life cycle be established in sub-Saharan African laboratories, this raises the possibility of undertaking dual *Bm* and *Onchocerca* macrofilarial implants within the same host, which would powerfully address the issue of drug effect on different filarial genera.

Given that SCID mice have an impaired mechanism of foreign tissue rejection and are routinely used for grafting human tumours [30] it was hypothesized that *O. ochengi* onchocercomata from the dermis of infected cows could survive upon engraftment into these mice, giving rise to the possibility of developing a murine model containing adult males, females and mf and keeping the natural anatomical structure of the parasitic niche intact. This would offer a desirable refinement to the male *Onchocerca* SCID implant model and control for testing drug exposure effects at a more naturalistic site of sub-cutaneous parasitism and/or emulate drug targeting of worms within a complex tissue surrounding of the onchocercoma. For this reason we undertook a pilot study whereby onchocercomata were implanted both into the peritoneal cavity and sub-cutaneously. It was observed that, at both sites, many of the nodules had become attached to host tissues and the majority of nodules had evidence of rapid host neovascularization. Vascularisation with both blood and lymphatic vessels is a consistent feature of *O. volvulus* nodule microarchitecture [50-52]. Whether neovascularisation is actively induced by pro-angiogenic parasite secretions [53] or is part of an innate inflammatory response to foreign material, remains to be resolved. Analogous to solid tumours, that can be successfully targeted by antiangiogenic therapies, neovascularisation is possibly a pre-requisite for more protracted survival, as a source of nutrients and oxygen supply deep within the nodule. Whilst only evaluated for seven days, our pilot data is encouraging; 83% of the implanted nodules contained motile female macrofilariae and 25% also yielded motile male macrofilariae *ex vivo*. Whilst no mf were observed from the culture of skin from the ear or tail in SCID recipients of sub-cutaneous nodules, motile mf were liberated after culturing recovered implants*.* This suggests that female macrofilariae are fertile and producing mf post-implant *in vivo*. Because *O. lienalis* mf show a cumulative increase in recruitment to the skin during the first three weeks following inoculations in SCID mice [22] extended experiments are required to determine the dynamics of mf recruitment to the skin from implanted nodules. As lymphatic growth into nodular tissue has been proffered as an exit route for mf migration into the skin [52], this process may be dependent on lymphangiogenic responses developing within implanted nodules.

These experiments are ongoing and require further optimization and refinement to test whether survival can be achieved for longer periods *in vivo*, and thus whether an onchocercoma xenograft (OX) model could also serve as a platform for the screening of novel therapeutics. Rajan *et al.* previously described that exposed ‘loops’ of *O. volvulus* female worms embedded in onchocercomata could remain viable and contain developing mf for up to 20 wks post-implant in SCID mice [20], demonstrating the long-term feasibility of this approach. We speculate that the reduced biomass and less dense extra-cellular matrix of *ochengi vs. volvulus* onchocercomata may facilitate an increase in perfusion of host solutes, as well as our observations of neovascularization, to support protracted survival of implanted female macrofilariae.

The full development of *B. malayi* and the protracted survival of implanted *O. ochengi* in SCID mice raises the possibility that this research model might be exploited for drug screening against other medically important filariae. Recently, it has been demonstrated that *L. loa* can develop to adulthood in immunodeficient mice (BALB/c IL-4Rα^−/−^/IL-5^−/−^), although patent infections producing circulating mf were not achieved [54]. Because the availability of a *L. loa* microfilaraemic mouse model would supplement the identification of safe macrofilaricides against onchocerciasis, an ongoing area of investigation is the testing of *L. loa* development and survival within inbred SCID mouse lines.

## Conclusions

SCID mice can be successfully utilised to maintain all life cycle stages of the lymphatic filariae, *Brugia malayi* and adult stages of the cattle filaria, *Onchocerca ochengi* with protracted survival*.* A range of reference anti-filarial drugs including macrofilaricides targeting nematode or *Wolbachia* endosymbionts have been tested against *Bm* and *O. ochengi* in SCID mice. These reference drugs perform with matching efficacy compared with either immune-competent controls or between the two filarial genera. Thus, we have established a ‘pan-filarial’ *in vivo* pre-clinical tool suitable to screen novel macrofilaricides against both lymphatic and *Onchocerca* filarial genera of medical importance.
